# Wounds of the Liver

**Published:** 1906-12

**Authors:** John Egerton Cannaday

**Affiliations:** Surgeon-in-Charge of Sheltering Arms Hospital, Hansford, W. Va.


### WOUNDS OF THE LIVER.*

By JOHN EGERTON CANNADAY, M.D.,

Surgeon-in-Charge of Sheltering Arms Hospital, Hansford, W. Va.

By virtue of its large size and somewhat friable character, the liver, in spite of its sheltered position under the diaphragm and surrounded by the bony and cartilaginous chest wall, is quite easily wounded by different forms of violence. The most common causes of liver traumatism are stab wounds, gunshot wounds and various forms of external violence by which the rupturing or tearing force is transmitted through the chest wall. The kick of a horse, a blow from a falling piece of timber, the force of an explosion from a premature shot in a mine, compression between two mine cars or railway accidents are among the commoner causes.

The lower border and anterior surface of the organ is the part most usually injured. The bleeding from such wounds is always profuse.

The symptoms, while not characteristic, are in general those of internal hemorrhage, and are fairly typical and constant. There is a gradually increasing pulse-rate, tenderness over the injured area, the abdominal muscles, especially those of the affected side, are board-like in their rigidity, due to nature's efforts to secure immobility for the injured part. A temperature rise and symptoms of peritonitis develop later.

The outlook for these cases, without early treatment, is exceedingly grave; with proper surgical intervention sixty or seventy per cent. should recover. From the highly vascular character of the organ, bleeding will be severe and continuous. The fact that liver injuries are usually complicated by injuries of other viscera adds to the mortality.

Early laparotomy is urgently indicated. A vertically placed incision at the right border of the right rectus muscle will give good exploratory access. The danger of hernia from incision

*Read before the Fayette County Medical Society, Montgomery, W. Va., October 19, 1906.

at this point is practically nil. A good-sized sand-bag transversely placed under the patient's back brings the liver downward and forward and renders it more easy of access. The extent of the injury should be noted and steps taken to control the liver bleeding. The most satisfactory means of doing this is by the introduction of small catgut sutures with a needle having no cutting edges. These sutures should be placed parallel to the torn surface of the liver, should not be inserted near the edge of the injured part, should include a good-sized mass of tissue in their grasp and should be tied with the least degree of tension that will control the oozing of blood, or the sutures will cut through the friable tissues. It has been recently suggested that by making a V-shaped section in the raw border, one can easily approximate the edges and thus leave no denuded areas. If the lower anterior portion of the liver is injured, it can easily be reached and treated through the described incision, but if the injury is high up under the costal wall, resort will have to be made to the osteoplastic flap, as suggested by Dr. Willy Meyer, of New York. This method simplifies and makes easy what would otherwise be a difficult or impossible undertaking. Packing to control liver bleeding is a most unsatisfactory method and should not be resorted to if ligation is possible. The end of the packing strip will necessarily have to be carried through the wound in the parietes as a drain and will carry infection inward quite as rapidly as it drains serum outward. The free blood in the abdomen should be removed by dry sponging and the operation wound closed without drainage. I may say in passing that gunshot wounds of the liver are nearly always complicated by perforation of some of the hollow viscera, the stomach being the viscus most frequently injured, and upon the proper and prompt treatment of these injuries, as well as those of the liver, will the successful outcome of the case depend. I will briefly report six cases that have come under my notice during the past year:

CASE I.—Referred by Dr. E. B. Friedenwald of Eskdale. A young white man of splendid physique was shot through the left lobe of the liver, the pistol-ball ranging backward and to the left, passing through the stomach near its cardiac orifice. On account of the comparatively inaccessible location the stomach perforations were sutured with difficulty. The liver hemorrhage was very free and was controlled by gauze packing. This was removed the third day; septic symptoms supervened and the patient died at the close of the sixth day. On postmortem examination it was found that the portion of the liver lying between the gunshot wound and the stomach, an area of at least three square inches in extent, had become gangrenous, evidently by injury to the arterial supply of the part. Operation was performed six hours after receiving injury. This man had been shot at night during a drunken brawl. His stomach was full at the time and the abdominal cavity was naturally contaminated. The postmortem examination showed that the seat of the most intense infection and inflammatory reaction had been in the vicinity of the drain, while the infection from the stomach wound and the necrosis of a portion of the liver were strong morbid factors in this case, yet there is no doubt in my mind but that a large amount of infection was carried in by way of the drain.

CASE 2.—Colored male, age 32, having pistol-shot wound over region of stomach, was brought to the hospital; patient complained of sharp pain in region of the stomach; had an in-increasing pulse-rate and wished to go to stool frequently. Median laparotomy performed two hours after receiving injury. The course of the bullet was through the anterior portion of the stomach, backward and to the right, passing in its course through the body of the liver. The stomach wounds were sutured and the liver bleeding was controlled by a light gauze pack placed in the bullet track and the end brought out as a drain through the upper part of the abdominal incision. Patient did well for a few days; drain was removed the third day; septic symptoms soon appeared and death occurred the eighth day. No postmortem could be obtained in this case.

CASE 3.—Referred by Dr. P. B. Pendleton, of Longacre. A young colored woman, while up in the mountains picking berries, was accidentally shot by her husband with a shotgun.

The entire load of small shot struck her abdomen just above the right iliac crest and ranged upward. The destruction of tissue was frightful; skin, muscle, fascia and parietal peritoneum had been stripped apart by the impact of the charge. Operation five hours after receiving injury. The incision extended from anterior superior iliac spine to costal margin. Thirty-eight separate and distinct perforations of small and large intestines, stomach and gall bladder were closed by suture. Just to the right of the gall bladder was a ragged V-shaped wound of the liver. One of the gun-wads and several bits of clothing were removed from this. The liver wound was cleansed and sutured as best I could; blood sponged from the peritoneal cavity. The condition of the patient was desperate, and saline solution was administered by hypodermoclysis, both during and after the operation; wound closed without drainage. The woman rallied and for a few days did fairly well. However, by the seventh day, she succumbed to the effects of septic absorption.

CASE 4.—Referred by Dr. Fisher, of Leewood. A middle-aged Italian, while drunk, was shot through the abdomen. Patient's condition bad; many symptoms of peritonitis. Operation thirty-six hours after receiving injury. Abdomen filled with semi-purulent fluid. Intestines matted in places, lymph flakes abundant. Two large perforations of the splenic flexure of the colon and two of the stomach were sutured, one of the latter being in the posterior wall, while the other involved the anterior wall of the stomach. The ball, after leaving the stomach, had passed through the anterior edge of the liver for a short distance. This wound was sutured so as to practically approximate the walls of the channel made by the bullet. All fluid in the abdomen was removed by dry sponging and the operation wound closed without drainage. A hypodermoclysis of saline solution was given and the patient put to bed. Two days later he ate largely of bread and cheese smuggled to him by a friend, got out of bed during the absence of the nurse from the ward and in other ways showed that he had been thoroughly imbued with the true American spirit of freedom from restraint during his brief stay in this country. All to no avail, however, for this patient made an absolute and speedy recovery.

CASE 5.—Referred to by Dr. J. S. Shafer, of Cannelton, W. Va., a young white man. Pistol-shot wound, penetrating body, ranging from right to left. Operation eight hours after receiving injury. The bullet had ranged from right to left through the anterior portion of the liver, tearing an ugly channel through that organ. A part of the thin anterior margin of the liver was nearly torn off; this was removed and the wound surface sutured with catgut so as to prevent bleeding, which had been rather free. The peritoneal toilet was made and the wound of the abdominal parietes closed. Convalescence was uneventful.

CASE 6.—Referred by Dr. F. R. Wheelock, Mucklow, W. Va., An Italian thirty years of age had been injured by a premature shot, a large chunk of coal striking him in the side and back. As the man had considerable hematuria, rupture of the kidney was suspected. The patient was kept quiet in bed as far as possible. The hematuria diminished somewhat, but the pulse-rate was accelerated. Operation was determined on. I made Pean's transverse incision over the region of the right kidney. The kidney was carefully examined; the capsule was unbroken and I decided that the trouble was elsewhere. The parietal peritoneum was opened just beyond the insertion of the ascending mesocolon. The incision was carried upward to the region of the ninth costal cartilege, and downwards for two inches below the line of the transverse incision. The abdominal cavity was well filled with dark blood. A strip of the right lateral and anterior border of the liver was torn off with the exception of the end near the gall bladder, which was still attached. This strip was approximately two inches wide by four inches long. The attached end of the liver strip was severed, a few tension catgut sutures inserted and lightly tied to prevent bleeding, the abdomen cleansed and the enormous incision closed. The patient was given saline transfusion and put to bed. The man was unruly and got out of bed whenever an opportunity presented itself. He usually removed the dressing from the wound four or five times in twenty-four hours, but nevertheless made a rapid recovery and left the hospital on the thirteenth day, practically well.

CONCLUSIONS.

The very great importance of early and thorough investigation of intra-abdominal bleeding, exploratory abdominal section being made in case there is doubt.

The value of controlling liver hemorrhage by suture, making the peritoneal toilet by dry sponging instead of irrigation, and the avoidance of packing and drainage.

